# GLP2-2G-XTEN: A Pharmaceutical Protein with Improved Serum Half-Life and Efficacy in a Rat Crohn’s Disease Model

**DOI:** 10.1371/journal.pone.0050630

**Published:** 2012-11-26

**Authors:** Susan E. Alters, Bryant McLaughlin, Benjamin Spink, Tigran Lachinyan, Chia-wei Wang, Vladimir Podust, Volker Schellenberger, Willem P. C. Stemmer

**Affiliations:** Amunix Inc., Mountain View, California, United States of America; National Institutes of Health, United States of America

## Abstract

**Objectives:**

Glucagon-like peptide 2 (GLP2) is an intestinal growth factor that has been shown to stimulate intestinal growth and reduce disease severity in preclinical models of short bowel syndrome and inflammatory bowel disease. Teduglutide, a recombinant human GLP2 variant (GLP2-2G), has increased half-life and stability as compared to the native GLP2 peptide, but still requires twice daily dosing in preclinical models and daily dosing in the clinic. The goal of this study was to produce and characterize the preclinical pharmacokinetic and therapeutic properties of GLP2-2G-XTEN, a novel, long-acting form of GLP2-2G.

**Methodology and Results:**

A GLP2-2G-XTEN fusion protein with extended exposure profile was produced by genetic fusion of GLP2-2G peptide to XTEN, a long, unstructured, non-repetitive, hydrophilic sequence of amino acids. The serum half-life of GLP2-2G-XTEN in mice, rats and monkeys was 34, 38 and 120 hours, respectively. Intestinotrophic effects were demonstrated in normal rats, where GLP2-2G-XTEN administration resulted in a significant increase in both small intestine weight and length. Efficacy of the GLP2-2G-XTEN protein was compared to that of GLP2-2G peptide in a rat Crohn’s disease model, indomethacin-induced inflammation. Prophylactic administration of GLP2-2G-XTEN significantly increased the length, reduced the number of trans-ulcerations and adhesions, and reduced the TNFα content of the small intestine. GLP2-2G-XTEN demonstrated greater *in vivo* potency as compared to GLP2-2G peptide, and improvement in histopathology supported the GLP2-2G-XTEN treatment effects.

**Conclusions and Significance:**

GLP2-2G-XTEN is intestinotrophic and demonstrates efficacy in a rat Crohn’s disease model requiring a lower molar dose and less frequent dosing relative to GLP2-2G peptide. Allometric scaling based on pharmacokinetics from mouse, rat and monkey projects a human half-life of 240 hours. These improvements in preclinical pharmacokinetics and dosing indicate that GLP2-2G-XTEN may offer a superior therapeutic benefit for treatment of gastrointestinal diseases including Crohn’s disease.

## Introduction

Glucagon-like peptide 2 (GLP2) is a 33 amino acid peptide derived from the post-translational processing of glucagon. First identified by Drucker [1], GLP2 is secreted from the enteroendocrine L cells of the small intestine and colon in response to nutritional stimulation. A specific gastrointestinal growth factor, GLP2 has been shown to increase the ability of the intestine to digest and absorb nutrients. In addition, it was found to possess potent intestinotrophic properties as well as significant reparative activity for the mucosal epithelium of the small and large intestine [2]. These features indicate that the GLP2 receptor is an attractive target for development of treatment interventions to promote intestinal adaptation and repair.

Teduglutide (NPS Pharmaceuticals) is a recombinant human GLP2 variant with a stabilizing alanine to glycine substitution at the second amino acid residue (GLP2-2G). Preclinical studies have shown that GLP2, or GLP2-2G injection, causes an increase in intestinal length and weight, villus height, crypt depth and crypt cell proliferation in both normal rodents and during intestinal adaptation in surgical models of short bowel syndrome [1]–[7]. Clinical studies have confirmed the significant enterotrophic effects [8]–[11]. Teduglutide is pending approval by the US FDA and has received a recommendation for approval by the European Medicines Agency as treatment for short bowel syndrome.

GLP2 therapy may also be beneficial for treatment of other gastrointestinal diseases involving malabsorption, inflammation, or mucosal damage of the small intestine, including Crohn’s disease. Mucosal GLP2 concentrations are reduced in areas of colonic inflammation in mice with inflammatory bowel disease [12], and treatment with GLP2 or GLP2-2G has been shown to reduce inflammation and severity of disease in a variety of relevant models. These include indomethacin-induced enteritis [13], HLA-B27 [14], DSS- induced ulcerative colitis [15], [16], and TNBS-induced colitis [17]. In these models GLP2 or GLP2-2G reduced mucosal damage, crypt cell apoptosis, intestinal lesions, inflammation, and mucosal cytokine production including TNFα, IL-1β, and IFNγ.

Given the enterotrophic and anti-inflammatory effects of GLP2 in preclinical models, and the preferential location of GLP2 receptors within the areas of intestine most affected by Crohn’s disease [18], a clinical trial of Teduglutide in Crohn’s disease was undertaken. While further study is necessary, results from this study revealed that Teduglutide may be an effective therapy for inducing mucosal healing in patients with moderate to severe Crohn’s disease. In this pilot study, subjects received once or twice daily injections (dependent on body weight); the frequent injections were said to contribute to injection site reactions and high subject withdrawal rate, and it was suggested that future trials use a formulation that allows for less frequent injections [19]. Therefore, additional GLP2 therapeutics with better pharmacokinetic properties are needed.

Native GLP2 has a short half-life in human circulation of about seven minutes due to rapid proteolytic degradation by dipeptidyl peptidase IV (DPPIV) [20] as well as removal from circulation by kidney filtration. Both the intact and DPPIV cleaved forms are subject to renal clearance [21]. Teduglutide, the GLP2-2G variant, has increased resistance to DPPIV, and shows a modest increase in half-life to 3–5 hours in healthy humans [22].

In this study, we show that the GLP2-2G peptide genetically fused to XTEN yields a therapeutically active fusion protein with a greatly improved half-life and lower dose requirement in a rat Crohn’s disease model. XTEN is a long, unstructured, non-repetitive, hydrophilic sequence of amino acids [23], [24]. Fusion of XTEN to the C-terminus of GLP2-2G increases the hydrodynamic radius of the peptide, reducing glomerular filtration and increasing half-life. GLP2-2G-XTEN may offer a pharmacokinetic and therapeutic advantage over native GLP2-2G peptide for treatment of inflammatory bowel disease, including Crohn’s disease.

## Materials and Methods

### GLP2-2G-XTEN Sequence

HGDGSFSDEMNTILDNLAARDFINWLIQTKITDGGSPAGSPTSTEEGTSESATPESGPGTSTEPSEGSAPGSPAGSPTSTEEGTSTEPSEGSAPGTSTEPSEGSAPGTSESATPESGPGSEPATSGSETPGSEPATSGSETPGSPAGSPTSTEEGTSESATPESGPGTSTEPSEGSAPGTSTEPSEGSAPGSPAGSPTSTEEGTSTEPSEGSAPGTSTEPSEGSAPGTSESATPESGPGTSTEPSEGSAPGTSESATPESGPGSEPATSGSETPGTSTEPSEGSAPGTSTEPSEGSAPGTSESATPESGPGTSESATPESGPGSPAGSPTSTEEGTSESATPESGPGSEPATSGSETPGTSESATPESGPGTSTEPSEGSAPGTSTEPSEGSAPGTSTEPSEGSAPGTSTEPSEGSAPGTSTEPSEGSAPGTSTEPSEGSAPGSPAGSPTSTEEGTSTEPSEGSAPGTSESATPESGPGSEPATSGSETPGTSESATPESGPGSEPATSGSETPGTSESATPESGPGTSTEPSEGSAPGTSESATPESGPGSPAGSPTSTEEGSPAGSPTSTEEGSPAGSPTSTEEGTSESATPESGPGTSTEPSEGSAPGTSESATPESGPGSEPATSGSETPGTSESATPESGPGSEPATSGSETPGTSESATPESGPGTSTEPSEGSAPGSPAGSPTSTEEGTSESATPESGPGSEPATSGSETPGTSESATPESGPGSPAGSPTSTEEGSPAGSPTSTEEGTSTEPSEGSAPGTSESATPESGPGTSESATPESGPGTSESATPESGPGSEPATSGSETPGSEPATSGSETPGSPAGSPTSTEEGTSTEPSEGSAPGTSTEPSEGSAPGSEPATSGSETPGTSESATPESGPGTSTEPSEGSAPG.

### GLP2-2G Gene Construction and Expression

GLP2-2G-XTEN gene construction was performed as previously described [25]. The final construct comprised the gene encoding the cellulose binding domain (CBD) from *Clostridium thermocellum* (accession #ABN54273), a tobacco etch virus (TEV) protease recognition site (ENLYFQ), the GLP2-2G sequence, and an 864 amino acid XTEN sequence under control of a T7 promoter. The removal of CBD from GLP2-2G-XTEN is mediated by the TEV protease, which is co-expressed on the same plasmid but from a separate operon using the constitutive GroE promoter. The host strain for expression, AmE025, was derived from W3110 (Yale CGSC #4474) in which the *fhuA* gene was deleted by P1 transduction from strain JW0146-2 (Yale CGSC #8416) and the lambda DE3 prophage was integrated onto the chromosome using a λDE3 lysogenization kit (EMD Chemicals USA #69734). GLP2-2G-XTEN was expressed in a 5L glass jacketed fermentation vessel with a B. Braun Biostat B controller, with an initial growth temperature of 37°C, followed by a reduction to 26°C upon addition of IPTG at 20 hours run time. After a total fermentation run time of 45 hours the culture was harvested by centrifugation, yielding cell pellets ∼1 kg in wet weight. The pellets were stored frozen at −80°C until purification was initiated.

### GLP2-2G-XTEN Purification

The *E. coli* cell pellet was re-suspended in 20 mM Tri-HCl pH 7.5, 50 mM NaCl and lysed by homogenization. The resulting cell lysate was heat coagulated at 85°C for 10 minutes, rapidly cooled to 10°C and clarified by centrifugation. The supernatant was collected and stored at 4°C for purification. GLP2-2G-XTEN protein was purified to homogeneity out of the clarified, heat-treated lysate using three bind and elute chromatography steps, two anion exchange and one hydrophobic interaction, run on an AKTA FPLC system. The final material was formulated using diafiltration in 20 mM Tris-HCl pH 7.5, 135 mM NaCl, sterile filtered using a 0.22 micron filter, and frozen at −80°C until further use. Overall purification yield was approximately 30%.

### Analytical Size Exclusion Chromatography

Analytical size exclusion chromatography (SEC) was performed using a BioSep-SEC-s4000, 7.8 × 600 mm HPLC column (Phenomenex) connected to an LC2010 integrated HPLC system equipped with an autosampler and a UV/VIS detector (Shimadzu). The system and the column were equilibrated in 50 mM NaPO_4_ pH 6.5, 300 mM NaCl at a flow rate of 0.5 mL/min at ambient temperature. For column performance, a SEC column check standard (Phenomenex, AL0-3042) was used. For sample analysis, 20 µL of 1 mg/mL purified GLP2-2G-XTEN was injected and the absorbance at 214 nm was monitored for 75 min.

### ESI-MS

200 µg of purified GLP2-2G-XTEN protein was desalted by solid phase extraction using Extract-Clean C18 column (Discovery Sciences). Desalted protein solution in 0.1% formic acid, 50% acetonitrile was infused at 4 µl/min into a QSTAR XL mass spectrometer (AB Sciex). Multi-charge TOF spectrum was acquired in 800–1400 amu range. Zero-charge spectrum was obtained by Bayesian reconstruction in 10–100 kDa range.

### Peptide

Human GLP2-2G peptide (HGDGSFSDEMNTILDNLAARDFINWLIQTKITD) was purchased from American Peptide (catalog # - 304076). The purity was greater than 95% as confirmed by HPLC and MS.

### Potency Assay – GPCR

Sample analysis was performed by Millipore’s GPCRProfiler® service using Millipore’s cloned human GLP-2 Receptor -expressing cell line made in the Chem-11 host (Millipore catalog #HTS164C) and their standard assay conditions. Calcium flux was monitored in real-time by FLIPR analysis after addition of serial dilutions of GLP2-2G-XTEN and GLP2-2G. Calcium flux was monitored in real-time by FLIPR analysis after addition of serial dilutions of GLP2-2G-XTEN and GLP2-2G. Eight and four replicates of the assay were performed on GLP2-2G-XTEN and GLP2-2G, respectively. After baseline corrections were applied, percentage activation relative to the Millpore reference agonist Emax were calculated. Then, dose response curves and EC50 values were generated and calculated using GraphPad Prism (Sigmoidal Dose Response).

### Animal Ethics Statement

Animal welfare for these studies was in compliance with the U.S. Department of Agriculture’s (USDA) Animal Welfare Act (9 CFR Parts 1, 2 and 3). The Guide for the Care and Use of Laboratory Animals, Institute of Laboratory Animal Resources, National Academy Press, Washington, D.C., was followed. The contract facilities performing this work maintain an Animal Welfare Assurance statement with the National Institutes of Health, Office of Laboratory Animal Welfare. In order to ensure compliance, all protocols were approved by the Institutional Animal Care and Use Committee (IACUC) of each contract facility before the initiation of treatment. No procedures or test articles were used that would cause more than momentary pain or distress to the animals. Detailed protocols, in-life summaries, and study reports are on file at Amunix, Inc.

Monkeys completed a quarantine and acclimation period and only healthy animals were selected for study. All monkeys were housed individually in stainless steel cages and provided environmental enrichment during the study. Fluorescent lighting was provided via an automatic timer for approximately 12 hours per day. Food and water was available ad libitum. Temperature and humidity was monitored and recorded daily and maintained to the maximum extent possible between 64 to 84°F and 30 to 70%, respectively.

### Pharmacokinetics

Pharmacokinetic studies in mice were performed using female C57BL/6 mice dosed subcutaneously (3 mice per time point) with 25 nmol/kg (2 mg/kg) GLP2-2G-XTEN. Plasma samples were collected at pre-dose and at selected time points over 120 hours in heparinized collection tubes. Pharmacokinetic studies in rats were done using catheterized female Wistar rats (3 rats per group) dosed subcutaneously with 25 nmol/kg and 200 nmol/kg GLP2-2G-XTEN. Plasma samples were collected at pre-dose and at selected time points over 168 hours in heparinized collection tubes.

The pharmacokinetics of GLP2-2G-XTEN after intravenous and subcutaneous injection was determined in male cynomolgus monkeys. Monkeys (6 per group) were dosed with 25 nmol/kg GLP2-2G-XTEN by intravenous or subcutaneous administration using a cross-over study design. Plasma samples were taken pre-dose and at selected time points post-dose up to 21 days (504 hours) following injection in heparinized collection tubes.

### ELISA for Pharmacokinetic Experiments

A quantitative sandwich enzyme-linked immunosorption assay (ELISA) technique was developed to measure GLP2-2G-XTEN in mouse, rat and cynomolgus monkey plasma. In the assay, standards, controls and test samples were incubated with mouse anti-XTEN monoclonal antibody (ProSci, 4D9G3) which was immobilized on a microtiter plate at a concentration of 8 µg/mL. After incubation, unbound material was washed away and GLP2-2G-XTEN was detected using biotinylated rabbit polyclonal to human GLP2 antibody (Phoenix Pharmaceuticals, B-G-028) at a 1∶20,000 dilution, followed by streptavidin-HRP (Pierce, 21130) at 0.1 µg/mL, and visualized with a TMB peroxidase substrate (Immunochemistry Technologies, SUB1). The assay has a range of 480–0.47 ng/mL in 3% plasma.

### Analysis of Pharmacokinetic Experiments

Pharmacokinetic curves were analyzed by fitting a non-compartmental model to the profile from each animal. The resulting pharmacokinetic parameters were averaged for the final results. Bioavailability was determined by dividing the area under the curve (AUC_∞)_ from the subcutaneous administration and the AUC_∞_ from the intravenous administration on a per animal basis. The results were averaged for the final data. Analysis was performed using Phoenix WinNonLin software (Pharsight, Cary NC).

### Rat Intestinotrophic Studies

Small intestine growth in rats was measured as a primary pharmacodynamic endpoint. GLP2-2G peptide, GLP2-2G-XTEN or vehicle was administered via subcutaneous injection into male Sprague-Dawley rats weighing 200–220 grams (10–12 rats per group). GLP2-2G peptide was dosed using the previously published regimen of 12.5 nmol/kg (0.05 mg/kg) twice daily for 12 days. GLP2-2G-XTEN was dosed at 25 nmol/kg once daily for 12 days. After sacrifice, a midline incision was made, the small intestines were removed, stretched to their maximum length and the length recorded. The fecal material was flushed from the lumen and the small intestinal wet weight recorded.

### Indomethacin-induced Inflammation

Male Wistar rats (180–220 grams) were weighed and randomized into treatment groups of ten rats each to ensure balance for average body weight across the treatment groups. Using a prophylactic study design, rats were injected subcutaneously with vehicle, GLP2-2G peptide (12.5 nmol/kg twice per day) or GLP2-2G-XTEN (25 nmol/kg once daily, 25 nmol/kg every other day for a total of three doses, or 75 nmol/kg once, as indicated), starting three days prior to indomethacin administration on day −3. Rats were fasted for 12 hours before receiving the first subcutaneous injection of freshly prepared indomethacin (7 mg/kg) on day 0; a second injection of indomethacin was administered 24 hours later on day 1. For the remainder of the experiment rats had unlimited access to food and water. Rats were euthanized 24 hours after receiving the second dose of indomethacin on day 2.

Disease readouts for the indomethacin model were performed as follows: 24 hours after the last indomethacin injection, rats were injected intravenously with 1 mL 1% Evan’s Blue dye. Thirty minutes later rats were anesthetized and exsanguinated. A midline incision was made in the abdomen and the rats were scored for the presence of adhesions. The small intestine was removed and the length recorded. The intestinal lumen was flushed with sterile 0.9% sodium chloride for injection and the length of the ulcerated area recorded. The intestines were gently blotted to remove excess fluid and weighed. The small intestine was scored for adhesions and trans-ulcerations using the following scoring system: Adhesions: none = 0, mild = 1, moderate = 2, severe = 3; transmural ulceration: none = 0, few = 1, multiple = 2, continuous = 3.

### Histopathology

Rat small intestine samples consisted of a 3 cm section of proximal jejunum and a 3 cm section of mid-jejunum collected 15 cm and 30 cm from the pylorus, respectively. Samples were fixed in 10% neutral buffered formalin. Samples were trimmed into multiple sections without bias toward lesion presence or absence. These sections were placed in cassettes, embedded in paraffin, microtomed at approximately 4 microns thickness, and stained with hematoxylin and eosin (H&E). The slides were evaluated microscopically by a board certified veterinary pathologist and scored for villous height as well as infiltration/inflammation, mucosal atrophy, villi/crypt appearance, abscesses/ulceration. A 1 to 4 severity grading scale was used, where 1 = minimal, 2 = mild, 3 = moderate, 4 = marked/severe, reflecting the combination of the cellular reactions seen histopathologically.

### Statistics

Parametric variables (small intestine length, small intestine weight, TNFα levels) were compared using an ANOVA with a Tukey-Kramer post hoc test for individual pairwise comparisons using an overall alpha of 0.05. Non-parametric variables (adhesion score, ulceration score) were compared with the vehicle control using a Mann Whitney U test with a Bonferroni correction for the p-value to create an overall alpha of 0.05. Calculations were performed using JMP or Excel as appropriate.

## Results

### Expression, Purification and G-protein Coupled Receptor Assay

**Figure 1:**
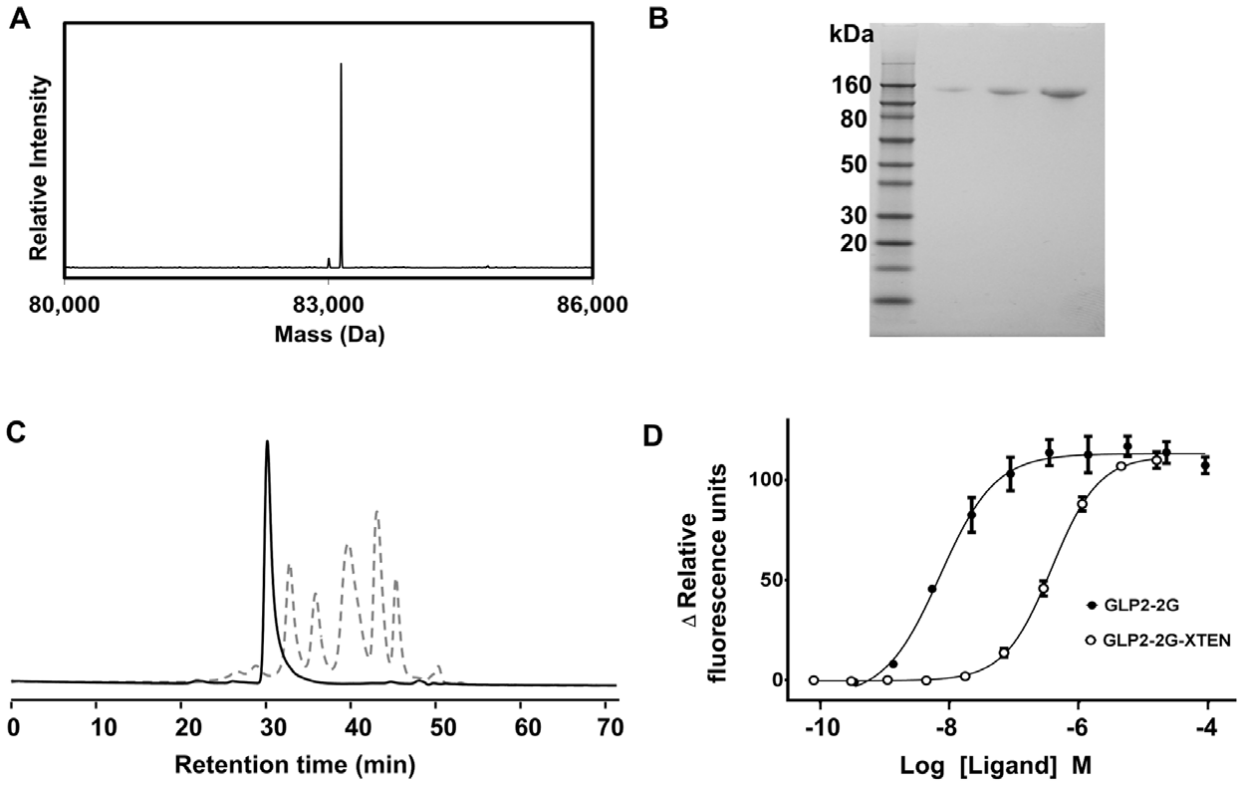
*In vitro* characterization of purified GLP2-2G-XTEN protein. A) ESI-MS analysis, major peak 83,142 Da – full length intact GLP2-2G-XTEN; minor peak 83,003 Da – des-His GLP2-2G-XTEN. B) non-reducing SDS-PAGE. Lane 1: molecular weight markers, lanes 2–4: 2 µg, 5 µg and 10 µg of GLP2-2G-XTEN, respectively. C) Size exclusion HPLC analysis. Solid curve: GLP2-2G-XTEN, 20 µg; dashed curve: molecular weights standard, which includes thyroglobulin 670 kDa, IgG 156 kDa, BSA 66 kDa, ovalbumin 45 kDa, myoglobin 17 kDa. D) Potency GPCR assay of GLP2-2G EC50 = 7 nM (95% confidence interval 4 nM to 12 nM) and GLP2-2G-XTEN EC50 = 380 nM (95% confidence interval 330 nM to 430 nM).

GLP2-2G-XTEN was expressed in *Escherichia coli* and purified to homogeneity (Figure 1; see Methods). Expression titers of 3–4 g/L were achieved for multiple batches with final purification yields of ∼30%. ESI-MS analysis of purified GLP2-2G-XTEN (calculated MW 83,144 Da) showed experimental molecular weight of 83,142 Da, with an accuracy of 24 ppm (Figure 1A). The only detectable impurity was a species lacking the N-terminal histidine, experimental molecular weight 83,003 Da (Figure 1A). Characterization of GLP2-2G-XTEN preparations by SDS-PAGE and size exclusion chromatography further indicates that the protein is homogeneous and non-aggregated in solution (Figure 1B and 1C). In a potency assay, based on calcium flux in a GLP-2 receptor expressing cell line, the purified protein and unmodified GLP2-2G were found to have EC50 values of 380 nM and 7 nM, respectively, indicating GLP2-2G-XTEN retains 2% of the potency (on a molar basis) as GLP2-2G peptide (Figure 1D).

### Pharmacokinetics

To assess the pharmacokinetics of GLP2-2G-XTEN, studies were performed in mice, rats and monkeys (Table 1). In all three species, animals were dosed subcutaneously with GLP2-2G-XTEN at 25 nmol/kg. Plasma levels of GLP2-2G-XTEN were determined at various time points using a sandwich ELISA with antibodies specific for the GLP2 and XTEN portions of the protein. The resulting pharmacokinetic curves were fit to a non-compartmental pharmacokinetic model and the terminal half-life of GLP2-2G-XTEN in mice, rats and monkey was determined to be 34, 38 and 120 hours respectively. In addition, the pharmacokinetic profile of GLP2-2G-XTEN after single subcutaneous administration to rats at 25 nmol/kg and 200 nmol/kg was dose proportional with the Cmax and AUC increasing in an approximately linear manner.

**Table 1:** Pharmacokinetic data for GLP2-2G-XTEN in mouse, rat and cynomolgus monkey.

Species	Route	Dose (nmol/kg)	T 1/2 (hour)	Cmax (ng/mL)	AUC_∞_ (hr*ng/mL)	Vd (mL/kg)	Clearance (mL/hr)
Mouse	SC	25	34	11,000	720,000	140	0.07
Rat	SC	25	38	6,900	530,000	210	0.80
Cyno	SC	25	120	20,000	3,400,000	110	2.0
Cyno	IV	25	110	62,000	3,700,000	90	1.9

**Figure 2:**
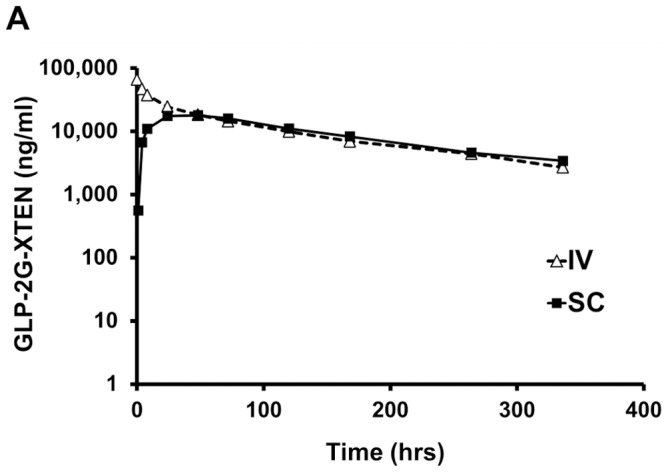
Pharmacokinetics of GLP2-2G-XTEN in cynomolgous monkeys. GLP2-2G-XTEN plasma concentration is shown following 25 nmol/kg administration via intravenous (triangle) or subcutaneous (square) route. Three animals were dosed by each route of administration. Data points are the average ± s.d. of three animals per time point.

To determine the bioavailability of GLP2-2G-XTEN after subcutaneous injection, the pharmacokinetics of the protein was studied in cynomolgus monkeys after intravenous and subcutaneous administration of 25 nmol/kg GLP2-2G-XTEN (Figure 2). The bioavailability was 96% demonstrating that GLP2-2G-XTEN is rapidly and near completely absorbed after subcutaneous administration.

### Rat Intestinotrophic Study

**Figure 3:**
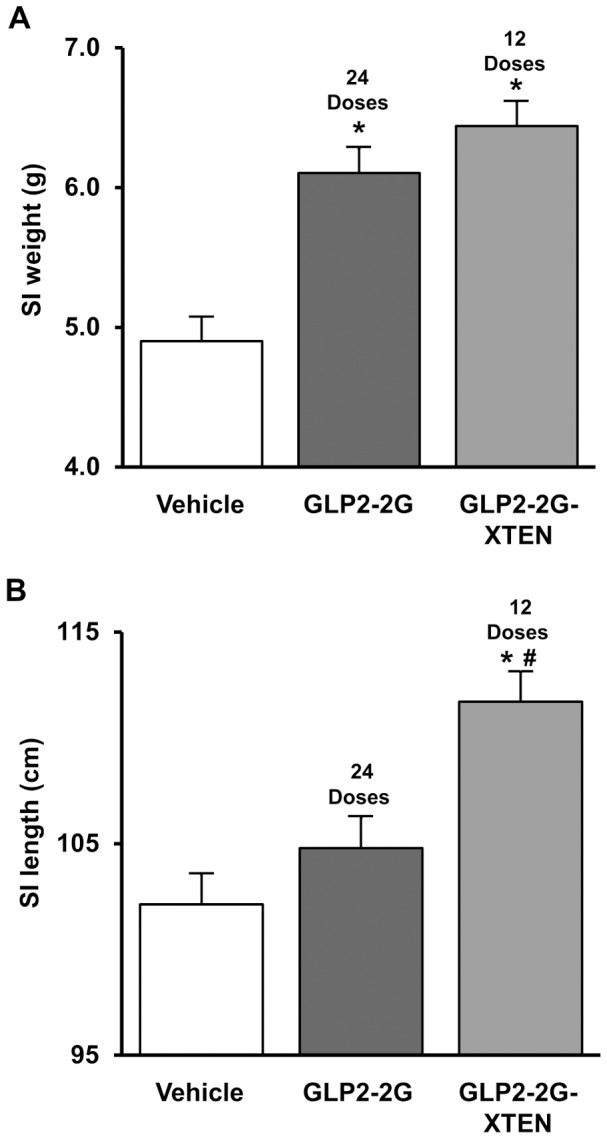
Intestinotrophic effects of GLP2-2G peptide and GLP2-2G-XTEN. Small intestine weight A) and length B) in normal rats following treatment with a total dose of 300 nmol/kg GLP2-2G peptide or GLP2-2G-XTEN. Data shown are from day 12 following SC injection of GLP2-2G peptide (12.5 nmol/kg per injection; twice per day for 12 days) and GLP2-2G-XTEN (25 nmol/kg per injection; once per day for 12 days). Data are means and SE, n = 10 per group. * P<0.05 compared to vehicle-treated rats, ^#^ P<0.05 compared to GLP2-2G peptide treated rats.

Intestinotrophic effects were assessed in normal rats administered GLP2-2G peptide and GLP2-2G–XTEN at doses previously shown to be efficacious in preclinical studies using GLP2-2G peptide [13]. Treatment with GLP2-2G peptide for 12 days (12.5 nmol/kg/dose using the standard twice daily dosing regimen) resulted in a significant increase in small intestine weight of 24% (Figure 3). There were no significant effects on small intestine length. Administration of equal molar GLP2-2G-XTEN over the 12 day study (25 nmol/kg/dose, once daily) resulted in a similar significant increase in small intestine weight of 31%. In contrast to the results seen with GLP2-2G peptide, the small intestine of GLP2-2G-XTEN treated rats showed a significant increase in length of 9% (10 cm), and was visibly thicker than the tissues from vehicle-treated control animals.

### Indomethacin-induced Disease Model

**Figure 4:**
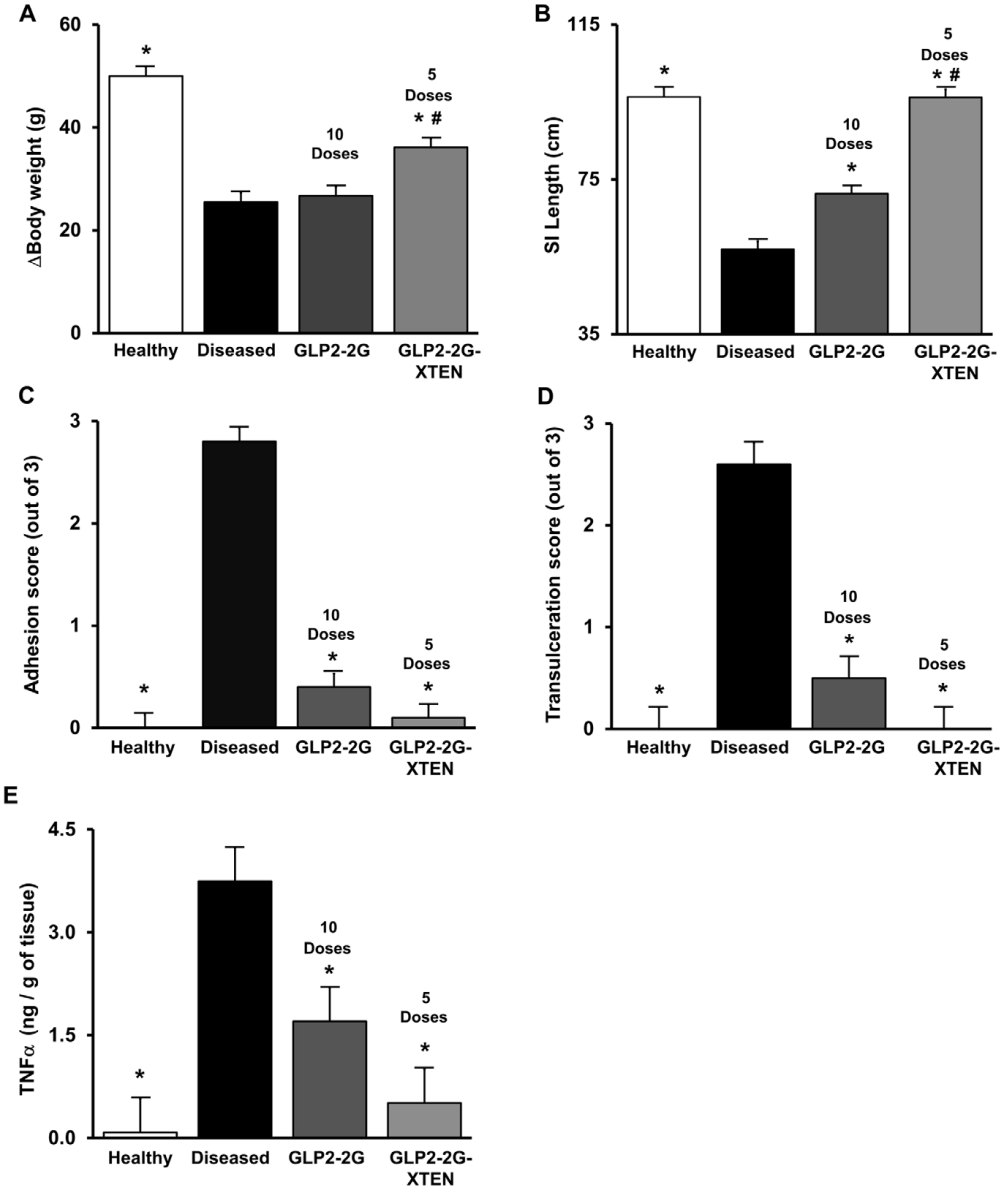
Efficacy of equal molar GLP2-2G-XTEN and GLP2-2G peptide in rat indomethacin-induced disease model. Data shown are A) change in body weight from day −3 through day 2, B) small intestine length, C) small intestine adhesion score, D) small intestine trans-ulceration score and E) small intestine TNFα concentration. Open bars are healthy rats treated with vehicle; colored bars are indomethacin-induced diseased rats treated with vehicle, GLP2-2G peptide (12.5 nmol/kg per injection; twice per day for 5 days) or GLP2-2G-XTEN (25 nmol/kg once per day for 5 days) as indicated. Compounds were dosed starting three days prior to first indomethacin injection (Day −3) and data shown are from the time of sacrifice on Day 2 (24 hours post second indomethacin injection). Data are means and SE, n = 10 per group. * P<0.05 compared to diseased, vehicle-treated rats, ^#^ P<0.05 compared to diseased GLP2-2G peptide treated rats.

GLP2-2G peptide has been studied in indomethacin-induced enteritis, a model for Crohn’s disease [13]. Indomethacin-induced disease was characterized by a significant reduction in weight gain relative to the non-diseased animals (Figure 4A). This reduction was accompanied by clinical signs including pale color, indicating possible anemia, poor body tone and discharge at the nose, indicative of distress, and black feces, indicative of occult blood. GLP2-2G peptide was administered twice a day for five days at 12.5 nmol/kg/dose (total dose of 125 nmol/kg), a dose regimen that demonstrated an intestinotrophic effect on small intestine weight and was shown to be effective at ameliorating symptoms in previous indomethacin-induced inflammation studies. For comparison, the efficacy of GLP2-2G-XTEN, was initially investigated using an equal molar dose (total dose of 125 nmol/kg) to that of GLP2-2G peptide over the course of the study. GLP2-2G-XTEN was administered once daily; GLP2-2G peptide was administered twice daily.

Administration of prophylactic GLP2-2G-XTEN starting three days prior to disease induction and continuing until the end of the study had a significant impact on the change in body weight; rats treated with GLP2-2G-XTEN showed a two-fold increase in net body weight gain versus vehicle treated diseased rats (Figure 4A). In contrast, treatment with GLP2-2G peptide had no significant effect on the change in body weight throughout the duration of the study.

As shown in Figure 4B–D, the small intestine of vehicle treated rats was severely damaged within 24 hours following the second indomethacin injection; it was significantly shorter in the vehicle treated diseased rats than in the non-diseased rats (56.9 vs.96.3 cm; Figure 4B) and 96% of the length of the small intestine was ulcerated. Examination revealed severe adhesions and trans-ulcerations (2.8 and 2.6 respectively out of a maximum score of 3). GLP2-2G-XTEN treatment resulted in a significant increase in small intestine length as compared to both vehicle-treated and peptide-treated rats, the small intestine length was close to that of normal, non-diseased rats. In addition, the ulceration and adhesion scores of GLP2-2G-XTEN treated rats also reverted back to normal, non-disease levels. In GLP2-2G peptide-treated rats, the small intestine was also significantly longer and less ulcerated than in the untreated diseased rats and GLP2-2G treatment resulted in a significant improvement in both adhesions and ulcerations although the levels did not return to those seen in normal, non-diseased rats.

Having documented GLP2-2G-XTEN intestinotrophic properties as well as protective effects on the small intestine mucosal epithelium, we next examined the anti-inflammatory potential of the molecule. Inflammation of the small intestine was determined by measuring the TNFα content by ELISA. As expected due to the inflammatory nature of the model, there was an approximately 50-fold increase in the TNFα content of small intestine in the vehicle treated indomethacin-induced diseased rats (∼3700 pg/g tissue) relative to the normal non-diseased tissue (∼80 pg/g tissue). Both GLP2-2G peptide and GLP2-2G-XTEN treatment significantly reduced the TNFα content of the diseased small intestine (Figure 4E), indicating an anti-inflammatory effect.

**Figure 5:**
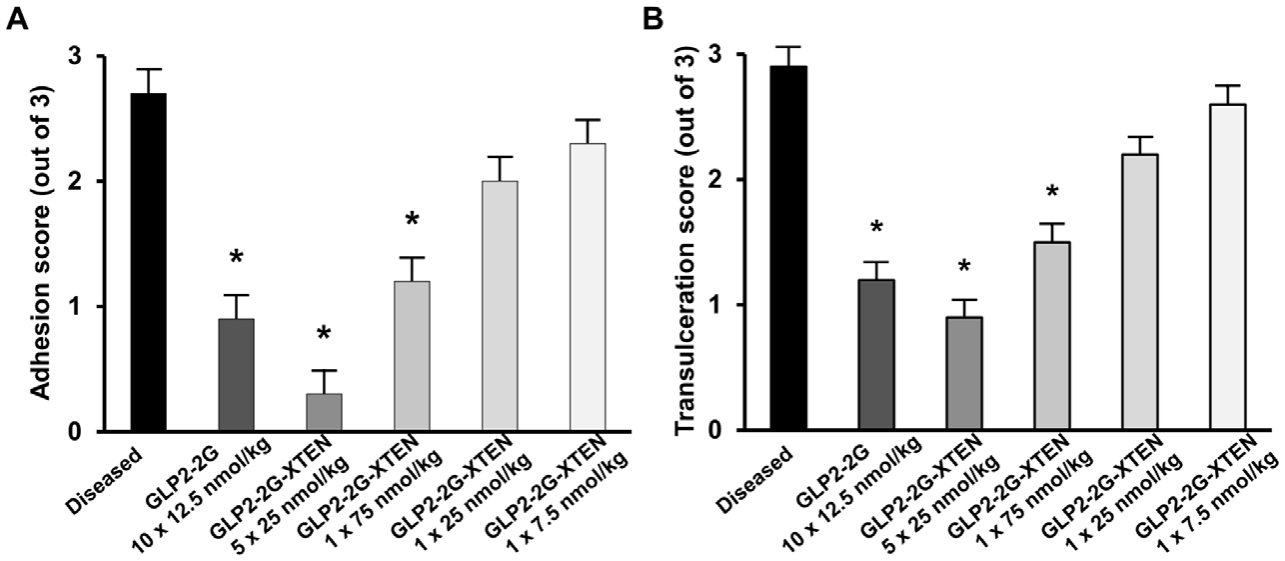
Efficacy of low dose GLP2-2G-XTEN treatment in rat indomethacin-induced disease model. Data shown are effects on A) small intestine adhesions and B) small intestine trans-ulcerations. Treatment with GLP2-2G peptide (12.5 nmol/kg per injection; twice per day for 5 days) or GLP2-2G-XTEN (25 nmol/kg once daily or only once at the indicated doses) was initiated on Day −3 and sacrifice is on Day 2. Data are means and SE, n = 10 per group. * P<0.05 compared to diseased, vehicle-treated rats.

**Figure 6:**
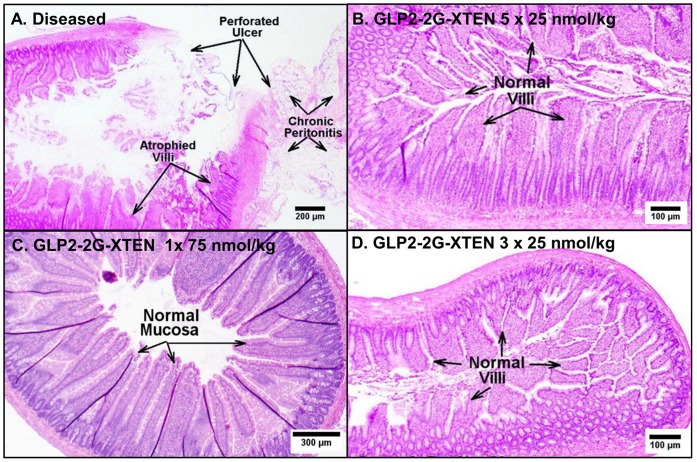
GLP2-2G-XTEN administration improves small intestine histopathology in the indomethacin-induced rat disease model. A) Hematoxylin and eosin staining of jejunum sections from A) diseased, vehicle-treated rats, B) diseased, GLP2-2G-XTEN high dose treated rats (125 nmol/kg total dose divided into five daily 25 nmol/kg doses), C) diseased, GLP2-2G-XTEN low dose treated rats (75 nmol/kg total dose given once on day −3) and D) diseased, GLP2-2G-XTEN low dose treated rats (divided into three 25 nmol/kg doses on day −3, −1 and 1).

**Figure 7:**
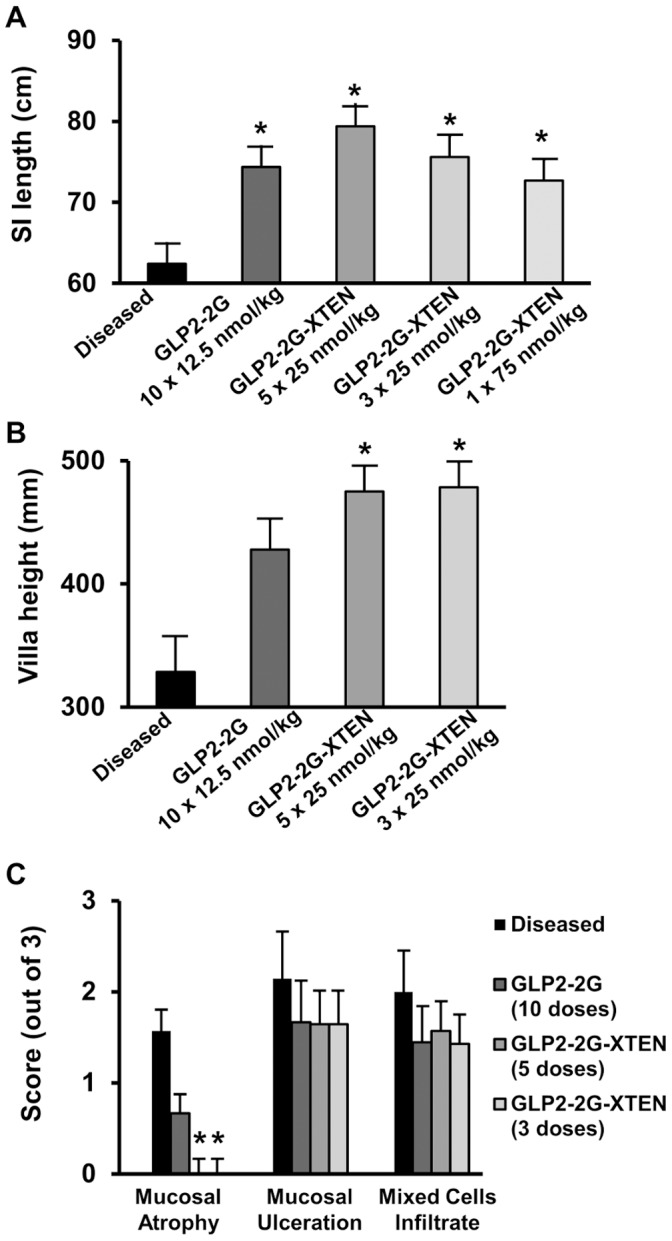
Treatment effects on small intestine length correlate with small intestine histopathology. A) Small intestine length B) villi height and C) mucosal atrophy, ulceration, infiltration measurements from diseased, vehicle-treated, GLP2-2G peptide-treated, and GLP2-2G-XTEN-treated rats. Quantitative histopathology was not performed on GLP2-2G-XTEN 75 nmol/kg single dose group.

To assess the *in vivo* potency of GLP2-2G-XTEN, dose ranging studies (Figure 5, 6, 7) were performed comparing lower doses of GLP2-2G-XTEN (total dose of 125 nmol/kg down to 7.5 nmol/kg administered as 5, 3, or 1 dose over the five day study) to the standard twice daily dose of GLP2-2G peptide (125 nmol/kg total dose administered as 10 doses). There was a clear dose-response to GLP2-2G-XTEN treatment (Figure 5). Furthermore, a single dose of 75 nmol/kg GLP2-2G-XTEN administered once on day −3 resulted in a significant reduction in adhesions and ulcerations, comparable to results seen using ten doses of GLP2-2G peptide at a total dose of 125 nmol/kg.

Histopathological analysis confirmed the GLP2-2G-XTEN treatment effects (Figure 6). Compared to normal rats, the ileum and jejunum of diseased rats showed severe mucosal atrophy, perforated ulcers, and chronic peritonitis; the villi appeared stunted and atrophied (Figure 6A). Treatment with GLP2-2G peptide (125 nmol/kg, ten doses, not shown), GLP2-2G-XTEN (125 nmol/kg, five doses, Figure 6B), and even low dose GLP2-2G-XTEN (75 nmol/kg, Figure 6C–D) showed a reduction in mucosal atrophy, ulceration, and infiltration as compared to untreated diseased rats; the villi appeared normal.

Quantitative histopathology was performed on a subset of samples. The increase in small intestine length of the GLP2-2G-XTEN-treated diseased rats as compared to vehicle-treated diseased rats (Figure 7A) correlated with a significant increase in villi height (Figure 7B). Both high (125 nmol/kg) and low (75 nmol/kg) dose GLP2-2G-XTEN-treated groups showed a significant increase in villi height; the increase in villi height seen in peptide treated rats was not significant. There was also a significant decrease in mucosal atrophy as both high and low dose GLP2-2G-XTEN-treated rats showed a significantly lower mucosal atrophy score than vehicle-treated diseased rats (Figure 7C). Although there was a trend showing a reduction in mucosal ulceration and mixed cell infiltrate following GLP2-2G-XTEN and GLP2-2G peptide treatment, these results were not significant for any of the three treatment groups.

## Discussion

Crohn’s disease is a chronic immune-related disorder of the gastrointestinal tract whose symptoms, including diarrhea, abdominal pain, weight loss and other complications, may lead to surgery, disability and increased mortality [19], [26]. There is no cure for Crohn’s disease and current therapy, targeted only toward the inflammatory component of the disease rather than providing for mucosal protection and healing, is not sufficient. These anti-inflammatory therapies, including an antibody to TNFα, show remission rates of only 26–38%, an increase of only 4.5–24% over placebo [27]–[31]. Therapy with agents targeting the GLP2 receptor provide for mucosal healing and repair and may increase the response rate in patients with Crohn’s disease. A pilot study revealed that Teduglutide induces mucosal healing and may be an effective therapy for patients with Crohn’s disease [19]. However, this pilot study also indicated that GLP2 therapeutics with better pharmacokinetic properties would be useful because the need for daily injections of Teduglutide led to a high withdrawal rate and placebo response.

The current study provides evidence that GLP2-2G-XTEN is a long-acting enterotrophic GLP2 receptor agonist that has increased *in vivo* exposure. GLP2-2G-XTEN increases the small intestine length and weight in normal rats and reduces the damage to the small intestine seen in a rat model of Crohn’s disease. Due to increased *in vivo* exposure, GLP2-2G-XTEN shows comparable therapeutic effects, including an increase in small intestine length, and a decrease in adhesions and ulcerations, when used at 60% of the total dose of GLP2-2G peptide. Furthermore, GLP2-2G-XTEN dosed once at 75 nmol/kg or three times at 25 nmol/kg is as effective as GLP2-2G peptide dosed ten times at 12.5 nmol/kg.

**Figure 8:**
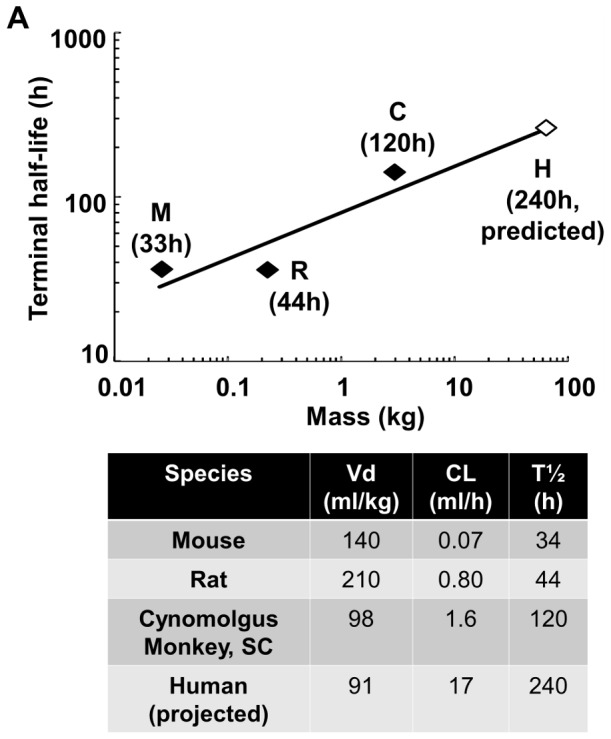
Allometric scaling human projection based on mouse, rat and monkey pharmacokinetic data. Allometric scaling was performed by fitting a linear regression model with the log of animal mass as the X variable and log of terminal half-life as the Y variable. The resulting linear model was extrapolated to a 70 kg human to produce a predicted value. The terminal half-life in humans can also be estimated using the predicted values for clearance (Cl) and volume of distribution (Vd) in the formula T½ = 0.693×Vd/Cl. Applying this formula yields a predicted terminal half-life of 240 hours in humans, which agrees with the linear extrapolation.

While it remains to be seen how the improvement in total molar dose and dosing frequency seen in a preclinical model of Crohn’s disease translates into the required clinical dose, the allometric scaling of GLP2-2G-XTEN half-life from three species reveals a projected human half-life of 240 hours (Figure 8). The robustness of this projection is bolstered by scaling the clearance (Cl) and volume of distributions (Vd) from the three species and calculating the terminal half-life (T½) using the relationship T½ = 0.693×Vd/Cl [32]. This results in a projection of 230 hours, in good agreement with the allometric scaling projection. Similar methodology was used to project the human half-life of the Ex4-XTEN fusion by Schellenberger et al [23]. The projection from the preclinical data was a human half-life of 139 hours, which is comparable to the actual half-life that was observed in the human trials (Diartis Pharmaceuticals, Inc., 2012 American Diabetes Association presentation). Therefore, the 240 hour projection is considered a reasonable estimate of anticipated human performance backed by a verified methodology.

This projected terminal half-life opens the possibility of monthly dosing intervals in humans, as the drug would pass through about three half-lives in a month. The peak to trough ratio is expected to be low, limiting safety concerns from the highest plasma concentrations, and hopefully increasing efficacy through a flatter, more even, exposure profile. The volume of distribution is greatly reduced compared to the unfused GLP2-2G peptide with the GLP2-2G-XTEN fusion being limited to approximately two times blood volume (Table 1) as is seen consistently with multiple XTEN fusions [23], [24]. Concentration of GLP2-2G-XTEN in the plasma implies a lower total dose requirement; however, because the volume of distribution is larger than blood volume, it also demonstrates that XTEN fusions are capable of penetrating into other peripheral tissues to reach sites of action.

In summary, our results demonstrate that prophylactic administration of GLP2-2G-XTEN, a long-acting GLP2 receptor agonist, increases small intestine length and reduces the severity of disease in a rat Crohn’s disease model. GLP2-2G-XTEN has increased *in vivo* exposure and requires a lower molar dose and less frequent dosing as compared with GLP2-2G peptide. Allometric scaling based on pharmacokinetics from mouse, rat and monkey projects a human half-life of 240 hours. GLP2-2G-XTEN may offer superior pharmacokinetics and a therapeutic benefit for treatment of gastrointestinal diseases involving malabsorption, inflammation or mucosal damage of the small intestine, including Crohn’s disease.
