# Correction: GLP2-2G-XTEN: A Pharmaceutical Protein with Improved Serum Half-Life and Efficacy in a Rat Crohn’s Disease Model

**DOI:** 10.1371/annotation/5c7138bd-5602-466d-8daf-e75e0b7d7fdb

**Published:** 2013-06-10

**Authors:** Susan E. Alters, Bryant McLaughlin, Benjamin Spink, Tigran Lachinyan, Chia-wei Wang, Vladimir Podust, Volker Schellenberger, Willem P. C. Stemmer

Figure 8 has an error in the value under "R". Please see the correct figure at the following link: 

**Figure:**
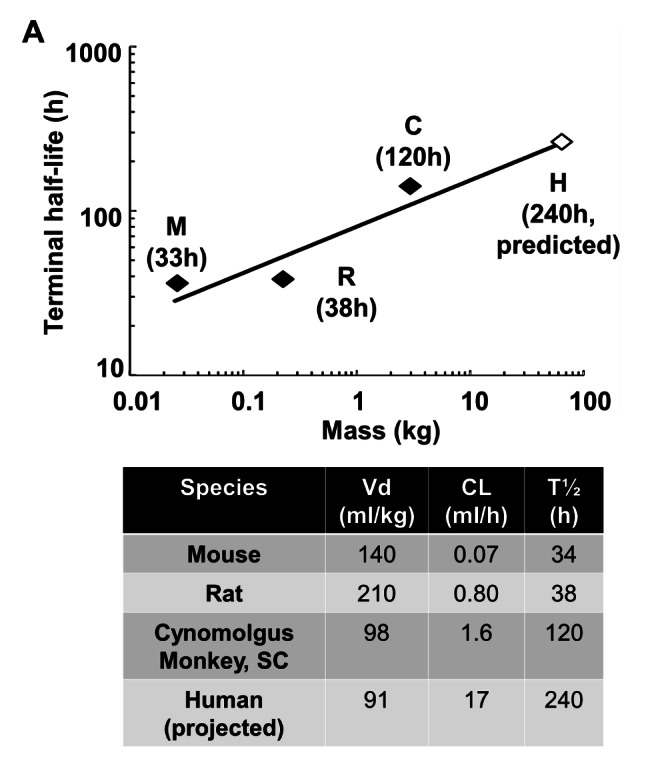


.

